# Remission of Proteinuria May Protect against Progression to Chronic Kidney Disease in Pediatric-Onset IgA Nephropathy

**DOI:** 10.3390/jcm9072058

**Published:** 2020-06-30

**Authors:** Jin-Soon Suh, Kyung Mi Jang, Hyesun Hyun, Myung Hyun Cho, Joo Hoon Lee, Young Seo Park, Jae Hyuk Oh, Ji Hong Kim, Kee Hwan Yoo, Woo Yeong Chung, Seong Heon Kim, Keehyuck Kim, Dae Yeol Lee, Jung Won Lee, Min Hyun Cho, Hyewon Park, Ja Wook Koo, Kyoung Hee Han, Eun Mi Yang, Keum Hwa Lee, Jae Il Shin, Heeyeon Cho, Kyo Soon Kim, Il-Soo Ha, Yong Hoon Park, Hee Gyung Kang

**Affiliations:** 1 Departments of Pediatrics, Bucheon St. Mary’s Hospital, College of Medicine, The Catholic University of Korea, Bucheon 14647, Korea; rebekahjs@hanmail.net; 2 Yeungnam University Hospital, Daegu 42415, Korea; fortune001j@gmail.com (K.M.J.); yhpark@medical.yu.ac.kr (Y.H.P.); 3 St. Vincent’s Hospital, College of Medicine, The Catholic University of Korea, Suwon 16247, Korea; inthy@naver.com; 4 Hallym University Sacred Heart Hospital, Anyang 14068, Korea; dong82dong82@naver.com; 5 Asan Medical Center Children’s Hospital, University of Ulsan College of Medicine, Seoul 05505, Korea; pedkid@gmail.com (J.H.L.); yspark@amc.seoul.kr (Y.S.P.); 6 Ajou University Hospital, School of Medicine, Suwon 16499, Korea; ssacktung@naver.com; 7 Department of Pediatrics, Yonsei University College of Medicine, Seoul 03722, Korea; KKKJHD@yuhs.ac (J.H.K.); AZSAGM@yuhs.ac (K.H.L.); SHINJI@yuhs.ac (J.I.S.); 8 Korea University Guro Hospital, Seoul 08308, Korea; guroped@korea.ac.kr; 9 Busan Paik Hospital, College of Medicine, Inje University, Busan 47392, Korea; chungwy@korea.com; 10 Pusan National University Children’s Hospital, Yangsan 50612, Korea; pedksh@gmail.com; 11 National Health Insurance Service Ilsan Hospital, Goyang 10444, Korea; kkim@nhimc.or.kr; 12 Jeonbuk National University Hospital, Jeonju 54907, Korea; leedy@jbnu.ac.kr; 13 Ewha Womans University Seoul Hospital, Seoul 07804, Korea; happymaniajw@hanmail.net; 14 School of Medicine, Kyungpook National University, Daegu 41944, Korea; chomh@knu.ac.kr; 15 Seoul National University Bundang Hospital, Seongnam 13620, Korea; ghyewon@gmail.com; 16 Inje University Sanggye Paik Hospital, Seoul 01757, Korea; koojw9@paik.ac.kr; 17 Jeju National University School of Medicine, Jeju 63243, Korea; hansyang78@gmail.com; 18 Chonnam National University Hospital and Medical School, Hwasun 58128, Korea; nuts99@naver.com; 19 Samsung Medical Center, Sungkyunkwan University School of Medicine, Seoul 06351, Korea; heeyeon1.cho@samsung.com; 20 Konkuk University Hospital, Seoul 05029, Korea; kimkyo@kuh.ac.kr; 21 Seoul National University College of Medicine and Seoul National University Children’s Hospital, Seoul 03080, Korea; ilsooha@snu.ac.kr

**Keywords:** children, IgA nephropathy, long-term outcome, remission of proteinuria

## Abstract

Immunoglobulin A nephropathy (IgAN) is one of the most common primary glomerulopathies diagnosed in children and adolescents. This study aimed to evaluate the clinical features in and outcomes of pediatric IgAN over the last 30 years. Patients who were diagnosed before age of 18 at 20 centers in Korea were evaluated retrospectively. Of the 1154 patients (768 males, 386 females) with a median follow-up of 5 years, 5.6% (*n* = 65) progressed to stage 3–5 chronic kidney disease (CKD). The 10- and 20-year CKD-free survival rates were 91.2% and 75.6%, respectively. Outcomes did not differ when comparing those in Korea who were diagnosed prior to versus after the year 2000. On multivariate analysis, combined asymptomatic hematuria and proteinuria as presenting symptoms and decreased renal function at the time of biopsy were associated with progression to CKD, while remission of proteinuria was negatively associated with this outcome. Patients who presented with gross hematuria or nephrotic syndrome tended toward positive outcomes, especially if they ultimately achieved remission. While remission of proteinuria might imply that the disease is inherently less aggressive, it also can be achieved by management. Therefore, more aggressive management might be required for pediatric-onset IgAN.

## 1. Introduction

Immunoglobulin A nephropathy (IgAN) is among the most common of the primary glomerulopathies in children and adolescents worldwide, especially in Asian countries [1]. The hallmark finding of IgAN is gross hematuria (GHU) in association with an upper respiratory infection, as was first described by Berger in 1968 [2]. However, more than half of the patients diagnosed with IgAN present with no subjective symptoms; the disease is frequently detected during the evaluation of asymptomatic urinary abnormalities (AUA), decreased renal function, or hypertension [3]. Along with variable initial presentations, IgAN has variable outcomes that include spontaneous remission, slow deterioration of renal function, or rapid progression to end-stage renal disease (ESRD) [4,5]. Therefore, efforts have been made to detect and to validate clinical and pathological risk factors that may be used to predict the outcome of and to determine treatments for patients diagnosed with IgAN [6,7,8].

Generally, renal outcomes with respect to pediatric-onset IgAN appear to be better than that those observed among those diagnosed with adult IgAN, although the results of long-term follow-up were inconsistent [8]. In a long-term observational study (mean follow-up of 18.7 years) of pediatric-onset IgAN patients in Finland, Ronkainen et al. [5] reported that 6 (11%) of 55 patients had developed ESRD at their last follow-up. In the Validation of the Oxford Classification of IgAN (VALIGA) study that included 174 children and adolescents diagnosed with IgAN from 13 European countries that were followed for a mean of 4.9 years, the survival rate given the combined endpoint of 50% decline in renal function or ESRD at 15 years was 94% [9]. Furthermore, Le et al. [10] reported that during a mean follow-up of 4.7 years, 24 (12.4%) of 218 children diagnosed with IgAN in China developed ESRD or 50% decline in renal function. In a Japanese study, Nozawa et al. [11] reported that after a mean follow-up of 7.3 years from onset, 7 (3.9%) of 181 pediatric IgAN patients developed ESRD with a predicted survival rate of 92.3% at 10 years and 89.1% at 20 years. Several clinical factors have been identified as risk factors for disease progression including persistence of a high level of proteinuria, higher time-averaged blood pressure during follow-up, and reduced renal function at the time of biopsy; pathologic variables identified as risk factors for IgAN include mesangial hypercellularity, tubular atrophy and interstitial fibrosis, crescents, and segmental glomerulosclerosis [12].

More than a few questions remain to be answered with respect to IgAN in children. For example, it is not clear whether the outcomes associated with pediatric cases are indeed superior to those of adult patients; it might be a result of lead-time bias. Likewise, the risk factors that were identified as associated with disease progression may not be applicable to a specific population of interest. At this time, mass urine screening tests of school children are performed in several Asian countries, although it is not clear whether this effort results in any improvements with respect to outcomes associated with IgAN. Similarly, it is not very clear whether initial presentation with nephrotic syndrome (NS) or GHU, and/or achievement of remission has a favorable impact on overall prognosis.

To address some of these questions and to broaden our knowledge of this common glomerulopathy, we conducted a nation-wide study with the goal of evaluating the long-term outcomes associated with IgAN.

## 2. Materials and Methods

### 2.1. Patients

We initially identified 1355 cases of patients diagnosed with IgAN by renal biopsy before the age of 18 during the period from 1985 to 2015 who were followed at one of 20 centers in Korea by pediatric nephrologists of the Korean Society of Pediatric Nephrology. A total of 201 patients with insufficient clinical or laboratory information or who were followed for <1 years were excluded from the study. Patients diagnosed with IgAN secondary to systemic lupus erythematosus or to other conditions including chronic hepatitis, diabetes, or cancer were also excluded; a total of 1154 subjects were enrolled in the study. The data at the initial visit, at the time of renal biopsy, and at the final observation, including clinical, laboratory, and pathologic information, were collected and reviewed retrospectively. In Korea, school urine screening (SUS) tests have been conducted since 1998 [3]. Therefore, the mode of detection (by SUS or not) was also evaluated, and the outcomes of those presented before and after the year 2000 were compared. Treatment history was also reviewed.

This study was approved by the Institutional Review Board of Seoul National University Hospital (IRB No. 1501-041-639) and followed the rules of the Declaration of Helsinki.

### 2.2. Laboratory and Pathologic Findings

Proteinuria was defined as greater than or equal to 0.3 g/g of urine protein to creatinine ratio (PCR) or ≥ +1 (30 mg/dL) on a dipstick test of an early morning urine sample [13]. Mild proteinuria was defined as 0.3 g/g ≤ urine PCR < 1 g/g or +1 (30 mg/dL) to +2 (100 mg/dL) on the dipstick test. Heavy proteinuria was defined as urine PCR ≥ 1 g/g or +3 (300 mg/dL) to +4 (1000 mg/dL) on the dipstick test [6]. Hematuria was defined as five or more red blood cells in a high-powered field in a centrifuged urine specimen examined by light microscopy. NS was defined as urinary protein excretion ≥ 40 mg/m^2^/h as assessed by a 24 h urine collection or random urine PCR ≥ 2 g/g with a serum albumin < 2.5 g/dL, with or without edema [11]. Estimated glomerular filtration rate (eGFR) was calculated using the creatinine-based bedside Schwartz equation in subjects aged less than 18 years [14]. In subjects who were >18 years old, eGFR was estimated by the modified modification of diet in renal disease (MDRD) equation [15]. Of note, each center used somewhat different pathologic classifications for IgAN patients, especially before the introduction of Oxford classification. Therefore, we re-evaluated the classifications on the basis of renal biopsy reports and re-scored three critical variables including mesangial hypercellularity (M0/M1), endocapillary hypercellularity (E0/E1), and crescents (C0/C1/C2), as described in the Oxford classification [16,17]. Renal biopsy reports from 627 of the 1154 patients were available for re-scoring (54.3%) for mesangial hypercellularity (M), from 628 patients (54.4%) for endocapillary hypercellularity (E), and from 618 patients (53.6%) for crescents (C).

### 2.3. Definitions of Outcomes

The remission of proteinuria was defined as a urine PCR < 0.3 g/g or negative to trace protein on urine dipstick tests of early morning urine samples. The recurrence of proteinuria was defined as the urine PCR was ≥0.5 g/g or ≥ +1 in urine dipstick test that reappeared after remission. The renal outcome was divided into five categories as follows: no abnormality (eGFR ≥ 90 mL/min/1.73 m^2^ and no proteinuria), minor abnormality (eGFR ≥ 60 mL/min/1.73 m^2^ and/or mild proteinuria), persistent nephropathy (eGFR ≥ 60 mL/min/1.73 m^2^ and urine PCR ≥ 1 g/g or 3+ or 4+ in dipstick test), renal insufficiency (eGFR < 60 mL/min/1.73 m^2^), and ESRD (eGFR < 15 mL/min/1.73 m^2^ or requiring renal replacement therapy) [6]. No or minor abnormalities were considered as favorable overall outcomes; all other conditions were regarded as unfavorable outcomes. The primary endpoint was deterioration of renal functional to an eGFR < 60 mL/min/1.73 m^2^; this was considered as a diagnosis of chronic kidney disease (CKD) in this study [18,19].

### 2.4. Statistical Analyses

The data are presented as frequencies and percentages for categorical variables. Continuous variables with normal distribution are presented as mean ± SD, while those with skewed distribution are shown as the median and interquartile range (IQR). Comparisons of groups were performed using the χ^2^ test, ANOVA, Student’s *t*-test, or Mann–Whitney test according to the characteristics of the variables. CKD-free and ESRD-free survival was analyzed using the Kaplan–Meier method; survival differences were evaluated by the log rank test. The relationship between various parameters and CKD-free survival was assessed using Cox proportional hazard model in a step-wise fashion. Analyses were carried out using SAS (version 9.4; SAS Institute Inc., Cary, NC, USA).

## 3. Results

### 3.1. Presentation and Clinical Course

Data on patient presentation and clinical course are shown in Table 1. For the 1154 patients diagnosed with IgAN and enrolled in our study (male-to-female ratio = 2), the mean ages at the initial visit and at the time of biopsy were 10.75 years and 11.41 years, respectively. A total of 120 patients (10.4%) reported a family history of glomerulonephritis or ESRD. Initial presenting symptoms included GHU (583 patients, or 50.5%), NS (89 patients, or 7.8%), and AUA (482 patients, or 41.8%). Among those presented with AUA, 126 patients (10.9%) presented with isolated hematuria (IH), 57 patients (4.9%) presented with isolated proteinuria (IP), and 299 patients (25.9%) presented with combined hematuria and proteinuria (CHP). Among patients with AUA (*n* = 482), 421 patients (87.3%) were diagnosed with urinary abnormalities via the nation-wide SUS. At renal biopsy, mean eGFR was 95.73 mL/min/1.73 m^2^; 7% of these patients (*n* = 81) had an eGFR of <60 mL/min/1.73 m^2^. Heavy proteinuria was present in 37.2% of the patients (*n* = 430). Renal pathology included mesangial proliferation in 50%, endocapillary hypercellularity in 8%, and crescents in 27% of the patients for whom pathology reports were available. Of the 1154 patients enrolled in the study, 207 patients (17.9%) received no pharmacologic treatments, 368 patients (31.9%) were treated with renin–angiotensin system (RAS) blockers only, and 579 (50.2%) were treated with additional immunosuppressive drugs. Over the course of the study, 839 (72.7%) patients achieved remission of proteinuria; among these, proteinuria recurred in 239 patients (28.5%).

### 3.2. Outcomes at the Final Evaluation

**Figure 1:**
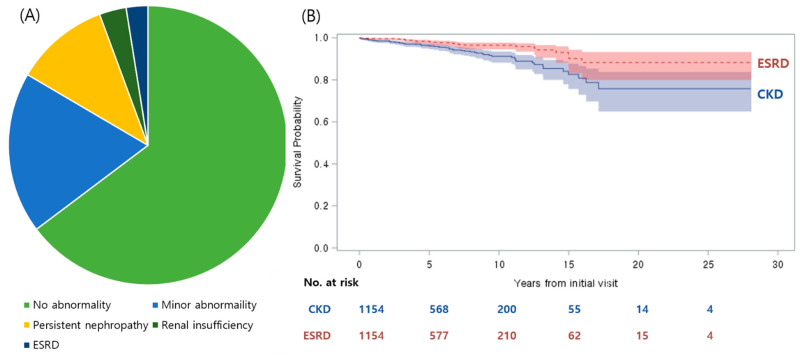
Final outcomes of Immunoglobulin A nephropathy (IgAN) patients at final follow-up visit. (**A**) Five categories of renal outcomes. (**B**) CKD–free and end–stage renal disease (ESRD)–free survival rates for all enrolled patients. CKD: chronic kidney disease stage 3–5.

Median duration from initial visit to final follow-up was 60 months (IQR 28–100.8 months), and the mean age at the final evaluation was 16.7 ± 5.6 years. At the final follow-up visit, 747 of patients (64.7%) had no residual abnormality, 216 patients (18.7%) had minor abnormalities, 126 patients (10.9%) were diagnosed with persistent nephropathy, 36 patients (3.1%) had renal insufficiency, and 29 patients (2.5%) were diagnosed with ESRD (Figure 1A); 191 patients (16.6%, male-to-female ratio of 1.1) had signs suggesting an unfavorable outcome and 65 patients (5.6%, male-to-female ratio of 1.6) were diagnosed with CKD (stage 3–5). The 5-, 10-, 15-, and 20-year CKD-free survival rates after the initial visit were calculated by the Kaplan–Meier method at 96.3% (95% confidence interval (CI), 94.8–97.4%), 91.2% (88.2–93.5%), 82.6% (75.7–87.7%), and 75.6% (64.9–83.7%), respectively (Figure 1B). For those ultimately diagnosed with CKD, the median time interval from initial visit to eGFR < 60 mL/min/1.73 m^2^ was 4.9 years (IQR 2.1–8.7 years), and the median age at primary endpoint was 19.08 years (IQR 15.9–22.9 years). By contrast, for those who progressed ESRD (*n* = 29, male-to-female ratio of 0.8), the median duration of follow-up was 4.3 years and the median age at ESRD was 15.5 years (IQR 13.8–20.6 years).

ESRD-free survival rates at 5-, 10-, 15-, and 20-years were 98.2% (95% CI, 97.0–99.0%), 96.5% (94.6–97.7%), 91.6% (85.5–95.2%), and 88.2% (79.9–93.2%), respectively (Figure 1B).

### 3.3. Clinical, Laboratory, and Pathological Parameters According to the Final Outcome

Clinical, laboratory, and pathological characteristics are as shown in Table 1. When patients with CKD (*n* = 65) were compared to those without CKD (*n* = 1089), the former were generally older at the time of renal biopsy and at the final follow-up visit; their follow-up duration was also longer (*p* < 0.001). There were no differences in family history, SUS as the mode of detection, presenting symptoms, or time of presentation (before or after 2000) between the two groups. Heavy proteinuria and lower eGFR at all three observational points were reported more commonly in those with CKD; likewise, serum albumin levels at both the initial and final visits were overall lower. In pathological findings, no differences were observed with respect to the proportions of M1/M0, E1/E0, or C0/C1/C2 when comparing results from the CKD to the non-CKD group. More patients diagnosed with CKD were treated with immunosuppressants (*p* < 0.001); the rate of remission was lower, and the incidence of relapse was higher among patients who were ultimately diagnosed with CKD (*p* < 0.001 for both).

When those in the favorable outcome group were compared to those in the unfavorable outcome group (i.e., those with persistent nephropathy or CKD), no differences in follow-up duration, family history, SUS as the mode of detection, or time of presentation were observed. The proportion of males was lower and the patients were older in the group with unfavorable outcomes. Those with unfavorable outcome frequently presented with AUA, notably, with CHP; GHU was observed less frequently (*p* < 0.001). Those with unfavorable outcome commonly presented with decreased renal function and heavy proteinuria. M1, E1, or crescents were more common in patients with unfavorable outcomes. Similar to CKD, despite the fact that patients in the unfavorable outcomes group typically were treated with immunosuppressive drugs, their remission rate was lower and their relapse rate was higher.

### 3.4. Comparisons of Parameters between Two Time Periods

**Figure 2:**
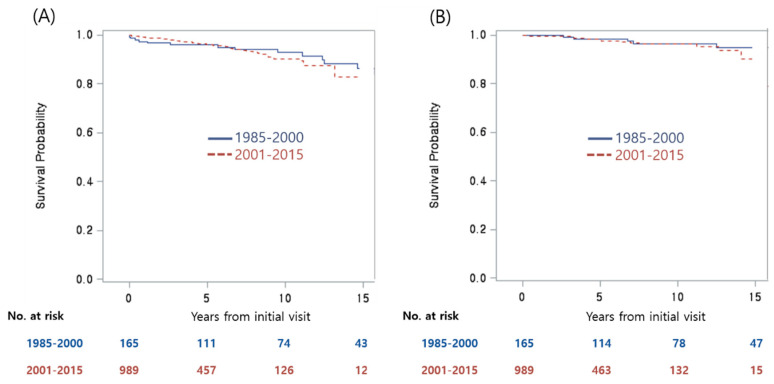
CKD–free (**A**) and ESRD–free (**B**) survival rates among patients presenting between 1985 and 2000 and those presenting between 2001 and 2015. CKD: chronic kidney disease stage 3–5; ESRD: end–stage renal disease.

Interestingly, the patients diagnosed between 2001 and 2015 were older at presentation and at subsequent renal biopsy than those from the earlier group; these more recent patients were also more likely to have a family history of glomerulopathy or ESRD. No differences in patient sex, remission, or relapse rate were detected (Table S1). As anticipated, presentation with AUA and via SUS was more common among the patients diagnosed in the more recent era. Similarly, renal function at all visits and serum albumin levels at initial presentation and at the time of renal biopsy were higher, and the proportion of patients with heavy proteinuria at renal biopsy were lower among those diagnosed between 2001 and 2015 than those in the 1985–2000 group; there were no differences in the proportions of patients with heavy proteinuria at presentation and at the final visit and serum albumin levels at final visit. M1 was less common and E1 were more common among patients diagnosed between 2001 and 2015, but the proportion of crescents did not differ. The more recent patients underwent more frequent treatment with immunosuppressive drugs, and remission rates, relapse rates, and final outcomes did not differ between the two groups. Importantly, the CKD-free or ESRD-free survival rates did not differ between the two time periods evaluated (i.e., 1985–2000 and 2001–2015; log rank, *p* = 0.252 and *p* = 0.249, respectively; Figure 2).

### 3.5. Risk Factors of Progression to CKD

**Figure 3:**
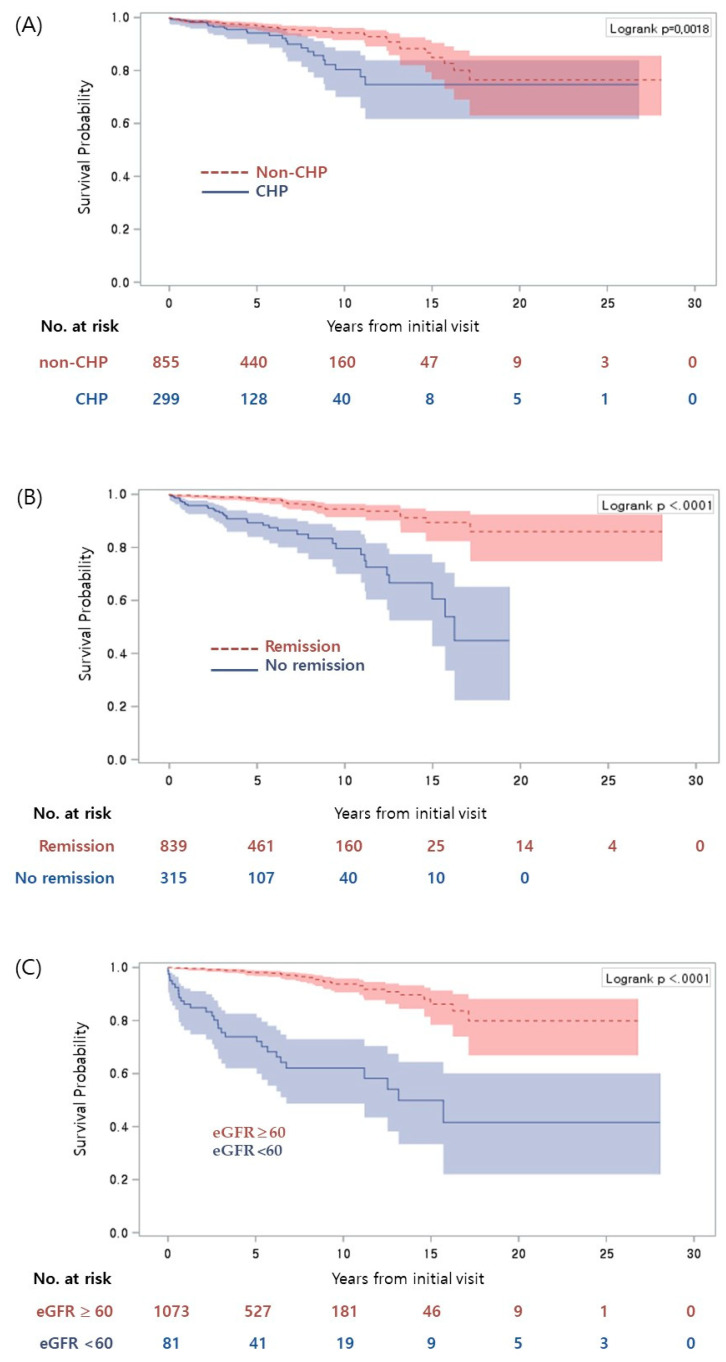
CKD–free survival rates with respect to three significant associated/independent factors. (**A**) Combined hematuria and proteinuria at presentation (CHP), (**B**) remission of proteinuria during the disease course, (**C**) estimated glomerular filtration rate <60 mL/min/1.73 m^2^ at renal biopsy. eGFR: estimated glomerular filtration rate, mL/min/1.73 m^2^.

Ten-year CKD-free survival rates were 95.8% for patients with GHU, 93.4% for IH, 78.7% for IP, 80.3% for CHP, and 93% for NS; 15-year survival rates were 84.4% for GHU, 74.7% for CHP, and 85.3% for NS. In a Cox regression analysis for evaluating risk factors that predict progression to CKD among IgAN patients, univariate analysis revealed that older age at presentation and at renal biopsy, CHP as a presenting symptom, decreased renal function (eGFR < 60 mL/min/1.73 m^2^) at presentation and at renal biopsy, and crescents (≥25%) on pathology were all associated with an increased risk of progression to CKD (stage 3–5). GHU as a presenting symptom and achieving remission during the course of disease were negatively associated with progression of CKD. In a multivariate analysis, CHP as a presenting symptom and decreased renal function (eGFR < 60 mL/min/1.73 m^2^) at renal biopsy remained significantly associated with progression of CKD, while achieving remission was an independent factor that was inversely correlated with progression of CKD (Table 2). CKD-free survival rates based on these significant independent factors are shown in Figure 3 and in Table S2.

**Figure 4:**
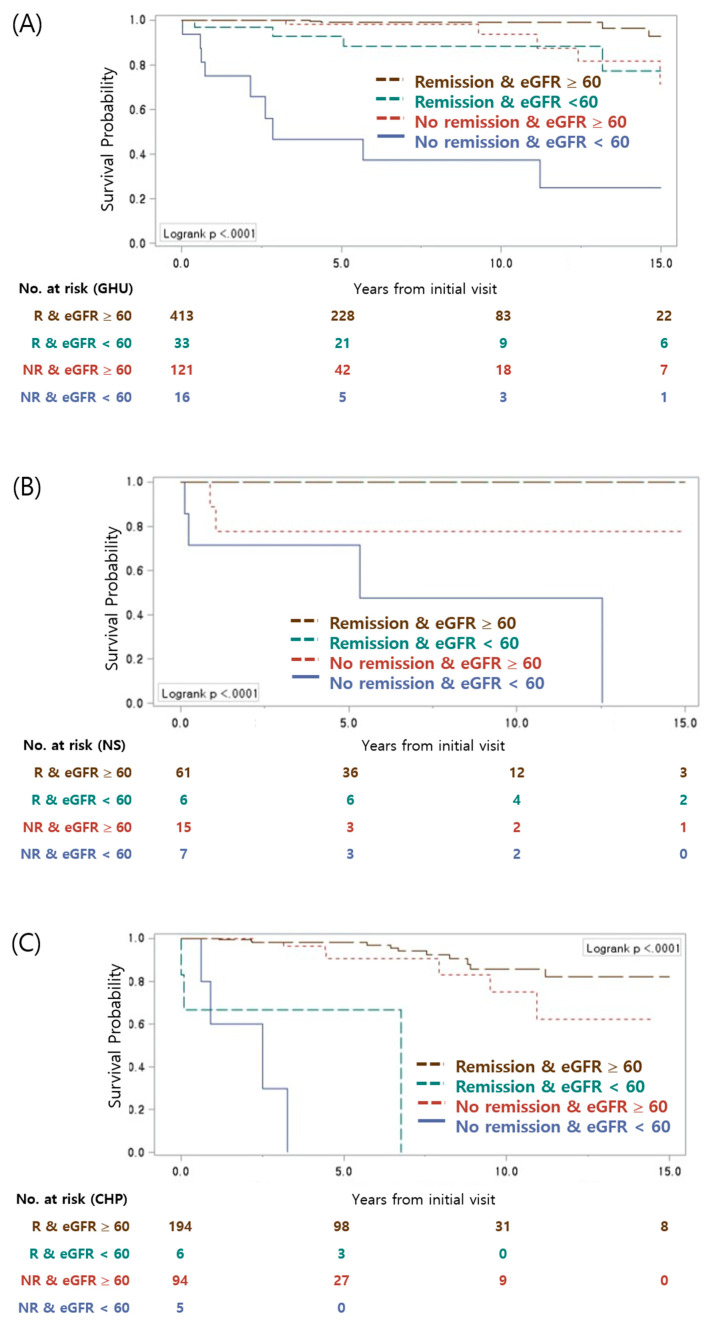
CKD–free survival rates among those who presented with (**A**) gross hematuria (GHU), (**B**) nephrotic syndrome (NS), or (**C**) combined hematuria and proteinuria (CHP) according to remission status or eGFR at renal biopsy. R: remission; NR: non-remission; eGFR: estimated glomerular filtration rate, mL/min/1.73 m^2^.

We further evaluated the outcomes of patients who presented with CHP, NS, or GHU according to remission status and eGFR at renal biopsy. These three presenting symptoms were selected because long-term outcomes were available (≥15 years). As shown in Figure 4 and Table S3, the CKD-free survival rates were significantly different depending on remission status or eGFR at renal biopsy, even in cases in which the patients had same presenting symptoms.

## 4. Discussion

The present study included 1154 patients and median observation period of 5 years. The overall rate of progression to CKD (stage 3–5) among pediatric patients in Korea who were diagnosed with IgAN was 5.6%; the 10- and 20-year CKD-free survival rates were 91.2% and 75.6%, respectively. These findings are similar to those reported in previous studies of pediatric-onset IgAN, which included a 10-year renal survival rate of 86–98% and a 20-year survival of 73–89% [20,21,22,23]. With respect to ESRD-free survival, the 10-year survival of 96.5% and 20-year survival of 88.2% were similar to analogous values reported in Japanese studies; interestingly, these rates were higher than those reported for European countries and for China [5,6,10,22,24]. These findings might reflect issues associated with patient ethnicity and/or the influence of the nation-wide mandatory school urinalysis screening that takes place in both Japan and Korea; mass screening facilitates early detection and management of IgAN [25]. Compared to adult IgAN patients of the same ethnicity, we observed ESRD-free survival rates that were higher among pediatric patients; Lee et al. [15] reported 10- and 20-year ESRD-free survival rates at 82% and 70.8%, respectively, for 1364 Korean adult IgAN patients who were treated during the years 1979 to 2008 and were followed during a median observation period of 96 months. Similarly, in a Japanese adult study, ESRD-free survival rates were 84.3% for 10-year and 66.6% for 20-year survival [26]. The better outcomes observed among pediatric IgAN patients might be the result of lead-time bias; longer-term follow-up data will be needed to compare survival rates in both pediatric-onset and adult-onset IgAN. Interestingly, 10% of total subjects had a family history of GN or ESRD. This finding might reflect the genetic predisposition to the development of IgAN, as shown in previous reports. However, it is difficult to compare the incidence of positive family history in IgAN patients with other cohorts because there are few reports evaluating family history of GN or ESRD in a large-scale cohort [27,28].

Characteristics of IgAN patients were quite different when comparing those diagnosed during the eras of before and after the year 2000. This outcome may be directly related to of the introduction of SUS. In Korea, SUS has been performed since 1998; IgAN was the most common of the findings from this screening test and from renal biopsies of patients presenting with AUA [3]. Although GHU still accounts for a considerable fraction of the initial presenting symptoms, more than 40% of the patients diagnosed in the more recent era presented with AUA. Compared with the patients diagnosed during the earlier era, patients diagnosed more recently presented with overall better laboratory findings and also underwent more frequent treatment with immunosuppressive drugs. These results suggested that, recently, patients in Korea who were diagnosed with IgAN patients were more likely to have been identified during the earlier stages of disease and to have had the opportunity to be managed with more active immunosuppressant therapy. Nevertheless, the CKD-free and ESRD-free survival rates did not differ significantly between the two eras, although the eGFR levels at the last evaluation were significantly higher among patients in recent era. By contrast, Japanese colleagues reported that ESRD associated with glomerulopathy improved after the introduction of SUS [25,29,30]. Considering that decreased renal function at diagnosis is an independent risk factor for progression to CKD progression, SUS may also help to improve outcomes of Korean IgAN patients via its capacity to identify patients at the early stages of disease.

Both CHP as a presenting symptom and eGFR < 60 mL/min/1.73 m^2^ at renal biopsy were found to be significant factors that were associated with progression to CKD. Our finding that decreased renal function at diagnosis is associated with poor outcome in patients diagnosed with IgAN is consistent with the results of previous studies [26,31]. CHP has also been identified as a risk factor for CKD progression in the general population [32,33]. In a long-term longitudinal study that targeted the general population of Korea (*n* = 8719), Kim et al. [32] reported that subjects with microscopic hematuria with proteinuria (*n* = 19) had a higher risk of CKD stage 3–5 than those who did not present with these clinical signs (Hazard Ratio 5.4, 95% CI 2.54–11.49, *p* < 0.001) during a median follow-up of 11.7 years. Moreover, in a Japanese study of adult patients with AUA initially identified as a result of mass urine screening (*n* = 772), a high proportion (23%) of individuals with CHP (*n* = 155) also presented with deterioration of renal function (serum creatinine > 2.0 mg/dL) during a median follow-up period of 6.3 years [33].Very recently, Shima et al. [34] reported that, among 25 children diagnosed with crescentic IgAN, 16 children (64%) were initially diagnosed with IgAN by SUS and most of them (14 of 16) presented with CHP. However, long-term outcomes of pediatric IgAN patients presenting with asymptomatic CHP are not known. To the best of our knowledge, this is the first study to determine that survival rates in patients presenting with CHP were lower than those with other presenting signs, including those first presenting with NS or GHU.

However, it is critical to recognize that not all patients who presented with these risk factors progressed to CKD. Among the 81 patients who presented with decreased renal function at biopsy and the 299 patients who presented with CHP, only 29 patients (35.8%) and 25 patients (8.4%), respectively, had progressed to CKD stage 3–5 at the final follow-up visit (Table S2). Therefore, other factors clearly have an impact on the final outcome. Analysis of risk factors revealed that achievement of remission during the course of disease provided significant protection against progression of IgAN to CKD; this is of importance, and this may be a clinically-modifiable factor that can be addressed with the goal of improving outcome, although remission of proteinuria might imply that the disease is inherently less aggressive. Likewise, NS was found to be a poor prognostic factor for IgAN patients in previous studies [35,36]; none of the IgAN patients in this study who presented with NS and achieved remission progressed to CKD stage 3–5. Several recent studies reported that the most significant factor with respect to survival is responsiveness to treatment [18,35]; the results of the present study also support this notion (Figure 4, Table S3).

For IgAN patients who presented with GHU, previous studies reported inconsistent outcomes [37,38]. In the present study, GHU was identified as a protective factor on univariate analysis; subjects who presented with GHU were among those who ultimately had more favorable outcomes than those who presented with IP, CHP, or NS. However, the CKD-free survival rates were significantly lower in patients with GHU who did not achieve remission; the 15-year survival rates (63.6%) among these patients were substantially lower than those of patients who presented with CHP who achieved remission (80.1%). Among those who presented with CHU, only 9 (2%) of 446 patients who achieved remission progressed to CKD; by contrast, 16 (11.7%) of 137 patients who did not achieve remission progressed to CKD. Therefore, responsiveness to treatment might be a significant factor with respect to survival among IgAN patients presenting with GHU, as well as among those who presented with NS or CHP (Figure 4). On the basis of these findings, we suggest that more aggressive therapeutic approaches are needed in order to achieve remission in the largest number of patients.

There are very few evidence-based guidelines for the use of immunosuppressants, including steroids, in children and adolescents diagnosed with IgAN [39]. Clinicians typically prescribe steroids when proteinuria is not reduced after several months in response to treatment with RAS blockers alone. As shown in this study, immunosuppressant treatment was more common among patients who progressed to CKD or among those with unfavorable outcomes, most likely because of non-responsiveness to RAS blockers. However, considering that remission is the most important prognostic factor and the time intervals from onset of disease to progression to CKD or ESRD is relatively short (4–6 years), immunosuppressant therapy might need to be initiated at an earlier stage of IgAN [40].

The other important factors that might have an impact on the outcome in IgAN patients are the pathologic variables. However, pathological findings, including mesangial hypercellularity, endocapillary hypercellularity, and detection of crescents were not significantly associated with outcome measures in this study; on the other hand, they did present significant risk with respect to unfavorable outcomes. This lack of statistical significance may relate to the limited availability of these critical data. Other limitations of this study include the absence of critical clinical information such as blood pressure measurements. As an estimating equation for eGFR for patients with age of >18 years, we used MDRD instead of the CKD epidemiology collaboration equation, which is more accurate if many of the subjects had high GFR [41]. In addition, we could not obtain comparable numbers of subjects from both the earlier and the later eras, and likewise, comparable follow-up durations when comparing CKD and non-CKD patients. As such, patient selection may have been biased. Nevertheless, the present study included a large number of pediatric-onset IgAN patients with a long-term follow-up.

## 5. Conclusions

The results of this study suggest that the outcomes in this cohort of Asian children diagnosed with IgAN were similar to those previously reported. Our nation-wide study with long-term observation revealed that CHP as a presenting symptom and decreased renal function at biopsy were both important risk factors for progression to CKD. Achieving remission of proteinuria was an independent prognostic factor that negatively correlated with progression to CKD; this is important given that this may be a clinically modifiable variable, while remission of proteinuria might imply that the disease is inherently less aggressive. Mass urine screening tests might be helpful for early detection and treatment; however, SUS as a mode of detection did not have a significant impact on the outcome of IgAN.

## Figures and Tables

**Table 1:** Comparisons of clinical, laboratory, and pathological parameters according to final outcome.

	Total	Favorable	Persistent N	CKD	*p1* *(3 groups)*	*p2* *(CKD* vs. *non-CKD)*	*p3* *(Favorable* vs. *Unfavorable)*
	(*N* = 1154)	(*N* = 963)	(*N* = 126)	(*N* = 65)			
**Demographics**							
Male sex	768 (66.55)	666 (69.16)	62 (49.2)	40 (61.54)	<0.001	0.3779	<0.001
Age at initial visit (years)	10.75 ± 3.75	10.57 ± 3.73	11.88 ± 3.62	11.24 ± 3.86	<0.001	0.1454	<0.001
Age at renal biopsy	11.41 ± 3.87	11.20 ± 3.89	12.44 ± 3.67	12.58 ± 3.37	<0.001	0.0056	<0.001
Age at last observation	16.69 ± 5.58	16.46 ± 5.63	16.60 ± 4.64	20.26 ± 5.41	<0.001	<0.001	0.002
FU duration * (months)	60 (28, 100.8)	59 (28, 99)	47 (20, 80)	97 (50, 160)	<0.001	<0.001	0.696
Family history (+)	120 (10.40)	94 (9.76)	23 (18.3)	3 (4.62)	0.0039	0.1159	0.119
SUS (+)	421 (36.48)	345 (35.83)	49 (38.9)	27 (41.54)	0.548	0.3833	0.324
Presenting symptoms							
Gross hematuria	583 (50.52)	503 (52.23)	55 (43.7)	25 (38.46)	0.0055	0.0645	<0.001
Isolated hematuria	126 (10.92)	114 (11.84)	8 (6.3)	4 (6.15)			
Isolated proteinuria	57 (4.94)	41 (4.26)	11 (8.7)	5 (7.69)			
Hematuria with proteinuria	299 (25.91)	236 (24.51)	38 (30.2)	25 (38.46)			
Nephrotic syndrome	89 (7.71)	69 (7.17)	14 (11.1)	6 (9.23)			
Year at presentation							
1985–2000	165 (14.30)	143 (14.85)	8 (6.3)	14 (21.5)	0.0086	0.0861	0.259
2001–2015	989 (85.70)	820 (85.15)	118 (93.7)	51 (78.5)			
**Laboratory findings**							
At initial visit							
eGFR (mL/min/1.73 m^2^)	93.54 ± 30.33	95.73 ± 28.36	94.39 ± 34.51	60.87 ± 30.29	<0.001	<0.001	<0.001
eGFR <60, *n* (%)	92 (8)	50 (5.19)	15 (11.9)	27 (41.54)	<0.001	<0.001	<0.001
Heavy proteinuria, *n* (%)	489 (42.37)	378 (40.26)	76 (60.3)	35 (53.85)	<0.001	0.05	<0.001
Serum albumin * (g/dL)	4.0 (3.5, 4.3)	4.0 (3.6, 4.4)	3.8 (3.1,4.2)	3.8 (3.3, 4.0)	<0.001	0.011	<0.001
At the time of renal biopsy							
eGFR (mL/min/1.73 m^2^)	95.73 ± 32.99	96.50 ± 30.02	106.59 ± 40.45	60.52 ± 34.54	<0.001	<0.001	0.183
eGFR <60, *n* (%)	81 (7.02)	42 (4.36)	10 (7.94)	29 (44.62)	<0.001	<0.001	<0.001
Heavy proteinuria, *n* (%)	430 (37.26)	313, 35.9%	78, 61.9%	39, 60%	<0.001	0.001	<0.001
Serum albumin * (g/dL)	4.0 (3.5, 4.3)	4.0 (3.6, 4.3)	3.7 (3.08, 4.1)	3.8 (3.3, 4.1)	<0.001	0.0757	<0.001
At last observation							
eGFR (mL/min/1.73 m^2^)	103.94 ± 35.48	110.49 ± 29.63	106.49 ± 26.21	31.41 ± 21.26	<0.001	<0.001	<0.001
Heavy proteinuria, *n* (%)	167, 14.47%	3, 0.31%	126, 100%	41, 63.08%	<0.001	<0.001	<0.001
Serum albumin * (g/dL)	4.4 (4.1, 4.6)	4.4 (4.2, 4.6)	4.0 (3.7, 4.3)	3.9 (3.5, 4.2)	<0.001	<0.001	<0.001
**Pathologic findings**							
M1	315 (50.24)	247 (48.05)	42 (57.5)	26 (65.00)	0.0493	0.0536	0.022
E1	53 (8.44)	35 (6.8)	14 (19.4)	4 (9.76)	0.0014	0.7691	0.004
Crescent							
None	451 (72.98)	384 (75.15)	45 (62.5)	22 (62.86)	0.0243	0.0563	0.012
<25%	133 (21.52)	104 (20.35)	21 (29.2)	8 (22.86)			
≥25%	34 (5.50)	23 (4.50)	6 (8.3)	5 (14.29)			
**Clinical course**							
Treatment							
None	207 (17.9)	203 (21.08)	3 (2.4)	1 (1.54)	<0.001	0.001	<0.001
RAS blocker	368 (31.9)	320 (33.23)	26 (20.6)	21 (32.31)			
Immunosuppressive drugs	579 (50.2)	440 (45.69)	97 (77)	43 (66.15)			
Remission rate	839 (72.7)	770 (79.96)	41 (32.5)	28 (43.1)	<0.001	<0.001	<0.001
Relapse rate	239 (28.5)	194 (25.19)	32 (82.1)	13 (46.4)	<0.001	<0.001	<0.001

Values are presented as number (%) or mean ± standard deviation unless otherwise indicated. * Values are presented as median (interquartile range, IQR). *p*1: comparison between the three groups of favorable outcome, persistent N, and chronic kidney disease (CKD); *p*2: comparison between CKD vs. non-CKD group (favorable + persistent N); *p*3: comparison between favorable vs. unfavorable (persistent N + CKD); M1: the score for mesangial hypercellularity >0.5; E1; the presence of endocapillary hypercellularity according to the Oxford classification for Immunoglobulin A nephropathy; CKD: chronic kidney disease stage 3–5, persistent N: persistent nephropathy; FU: follow-up; IQR: interquartile range; SUS: school urine screening; eGFR: estimated glomerular filtration rate; RAS blocker: renin–angiotensin–aldosterone blocker.

**Table 2:** Risk factors associated with progression to CKD.

	Univariate Analysis			Multivariate Analysis		
	*p*	HR	95% CI	*p*	HR	95% CI
Male/female	0.18	1.41	0.85–2.32			
Age at initial visit (per 1 year)	0.01	1.09	1.02–1.17	0.26	1.12	0.92–1.37
Age at renal biopsy (per 1 year)	0.008	1.09	1.02–1.16	0.8	0.98	0.80–1.19
Year at diagnosis						
1985–2000	Reference	1.46	0.76–2.80			
2001–2015	0.254					
Presenting symptoms						
Gross hematuria	0.014	0.532	0.32–0.88	0.4	1.39	0.53–3.64
Nephrotic syndrome	0.85	1.09	0.47–2.52			
Combined hematuria with proteinuria	0.0023	2.18	1.32–3.61	0.037	3.32	1.08–10.22
SUS	0.1	1.53	0.92–2.53			
Family history	0.21	0.47	0.15–1.51			
eGFR <60 mL/min/1.73 m^2^ at initial visits	<0.001	7.64	4.64–12.56	0.23	0.46	0.13–1.65
eGFR <60 mL/min/1.73 m^2^ at renal biopsy	<0.001	9.30	5.67–15.24	<0.001	18.49	5.51–62.06
Heavy proteinuria at initial visits	0.113	1.5	0.91–2.46			
Heavy proteinuria at renal biopsy	0.06	1.74	0.97–3.13			
M1	0.13	1.65	0.86–3.18			
E1	0.27	1.8	0.64–5.09			
Crescents ≥25%	0.02	3.19	1.23–8.33	0.95	0.96	0.27–3.47
Remission during disease course	<0.001	0.19	0.12–0.31	<0.001	0.12	0.06–0.26
Relapse after remission	0.13	1.85	0.83–4.14			

HR: hazard ratio, 95% CI: 95% confidence interval, SUS: school urine screening, eGFR: estimated glomerular filtration rate, M1: the score for mesangial hypercellularity >0.5, E1: the presence of endocapillary hypercellularity according to the Oxford classification for IgA nephropathy.

## Supplementary Materials

The following are available online at https://www.mdpi.com/2077-0383/9/7/2058/s1. Table S1: Comparisons of patient-associated parameters between two defined periods of time. Table S2: The proportions of patients progressing to CKD vs. CKD-free survival rates of patients based on independent risk factors associated with progression to CKD. Table S3: The proportions for CKD progression and CKD-free renal survival rates of patients presented with GHU, CHP, and NS according to the differences of renal function at biopsy and the presence of proteinuria remission.
