# Supplementary material for: Remission of Proteinuria May Protect against Progression to Chronic Kidney Disease in Pediatric-Onset IgA Nephropathy
Source: J Clin Med. 2020 Jun 30;9(7):2058. doi: 10.3390/jcm9072058 (PMC7408672; doi:10.3390/jcm9072058)
Supplement: Supplementary file 1 [file jcm-09-02058-s001.pdf]

Table S1. Comparisons of patient-associated parameters between two defined periods of time

	1985-2000 (N=165)	2001-2015 (N=989)	*p*
**Demographics**			
Male sex	115 (69.7)	653 (66)	0.374
Age at initial visit (years)	9.08 (7, 12.08)	11.08 (8.17, 13.83)	<0.001
Age at renal biopsy	9.33 (7.33, 12.33)	11.92 (8.67, 14.5)	<0.001
Age at last observation	18.58 (13.58, 24.08)	16.5 (12.92, 19.5)	<0.001
Family history (+)	10 (6.1)	110 (11.1)	0.049
SUS (+)	27 (16.4)	394 (39.8)	<0.001
Presenting symptoms			
Gross hematuria	95 (57.6)	488 (49.3)	<0.001
Isolated hematuria	6 (3.6)	120 (12.1)	
Isolated proteinuria	5 (3.0)	52 (5.3)	
Hematuria with proteinuria	33 (20)	266 (26.96)	
Nephrotic syndrome	26 (15.8)	63 (6.4)	
**Lab findings**			
At initial visit			
eGFR (mL/min/1.73m^2)	82.11 ± 28.17	94.93 ± 30.47	<.0001
eGFR <60, n (%)	26 (15.8)	66, 6.7%	<0.001
Heavy proteinuria	69, 41.82%	420, 42.47%	0.794
Serum albumin* (g/dL)	3.6 (3, 4)	4 (3.6, 4.4)	<0.001
At the time of renal biopsy			
eGFR (mL/min/1.73m^2)	84.87 ± 28.48	97.35 ± 33.33	<.0001
eGFR <60, n (%)	22, 13.3%	59, 6%	0.001
Heavy proteinuria	80, 48.48%	350, 35.39%	0.011
Serum albumin* (g/dL)	3.6 (3, 4.1)	4 (3.6, 4.3)	<0.001
At last observation			
eGFR (mL/min/1.73m^2)	94.86 ± 32.17	105.24 ± 35.77	<.0001
Heavy proteinuria	19, 11.5%	148, 15%	0.335
Serum albumin* (g/dL)	4.4 (4.1, 4.6)	4.4 (4.1, 4.6)	0.529
**Pathologic findings**			
M1	74 (71.8)	241 (46)	<0.001
E1	2 (1.9)	51 (9.7)	0.009
Crescent			
None	71 (71)	380 (73.4)	0.542
<25%	25 (25)	108 (20.8)	
≥25%	4 (4)	30 (5.8)	
**Clinical course**			
Treatment			
None	56 (33.9)	152 (15.4)	<0.001
RAS blocker	47 (28.5)	320 (32.4)	
Immunosuppressive drugs	62 (37.6)	516 (52.2)	
Remission rate	129 (78.2)	710 (71.8)	0.088
Relapse rate	27 (16.4)	212 (21.4)	0.082
**Outcome**			
No abnormality	109 (66.1)	638 (64.5)	0.966
Minor abnormality	34 (20.61)	182 (18.4)	
Persistent nephropathy	8 (4.85)	118 (11.9)	
Renal insufficiency	8 (4.85)	28 (2.8)	
ESRD	6 (3.6)	23 (2.3)	

Values are presented as number (%) or mean ± standard deviation unless otherwise indicated.

*Values are presented as median (interquartile range, IQR).

M1; the score for mesangial hypercellularity >0.5, E1; the presence of endocapillary hypercellularity according to the Oxford classification for IgAN

SUS; school urine screening, eGFR; estimated glomerular filtration rate, RAS blocker; renin-angiotensin-aldosterone blocker, ESRD; end-stage renal disease

Table S2. The proportions of patients progressing to CKD vs. CKD-free survival rates of patients based on independent risk factors associated with progression to CKD

	Number (%) of CKD progression	5-year survival (95% CI)	10-year survival (95% CI)	15-year survival (95% CI)	20-year survival (95% CI)	*P*
**CHP**						
Yes (n=299)	25 (8.4)	94.2% (90-96.6%)	80.3% (70-87.4%)	74.7% (61.7-83.9%)		0.0018
No (n=855)	40 (4.7)	97% (95.3-98.1%)	94.2% (91.5-96.1%)	84.9% (76.6-90.4%)	82.7% (73-89.1%)	
**eGFR at biopsy**						
≥60 (n=1073)	36 (3.4)	98.1% (96.7-98.9%)	93.8% (90.6-95.9%)	86.2% (78.4-91.3%)	80% (66.9-88.2%)	<0.001
<60 (n=81)	29 (35.8)	73.9% (62.0-82.6%)	62.1% (48.7-73.0%)	49.9% (33.4-64.4%)	41.6% (22.0-60.1%)	
**Remission achievement**						
Yes (n=839)	28 (3.3)	98.3% (97.0-99.1%)	94.4% (91.4-96.4%)	89.4% (82.3-93.7%)	85.9% (74.8-92.4%)	<0.001
No (n=315)	37 (11.8)	89.3% (83.9-92.9%)	79.6% (70-86.4%)	60.1% (42.7-74.4%)	44.9% (22.3-65.1%)	

CKD; chronic kidney disease stage 3–5, 95% CI; 95% confidence interval, CHP; combined hematuria with proteinuria as presentation, eGFR; estimated glomerular filtration rate

Table S3. The proportions for CKD progression and CKD-free renal survival rates of patients presented with GHU, CHP and NS according to the differences of renal function at biopsy and the presence of proteinuria remission

	**Number (%) of CKD progression**	**5-year (%)**	**10-year (%)**	**15-year (%)**	***p***
GHU					
Total (N=583)	23 (3.8)	97.2	95.8	84.4	
Group 1 (n=413)	4 (1.0)	99.2		92.7	<0.001
Group 2 (n=33)	4 (12.1)	93	88.6	77.5	
Group 3 (n=121)	5 (4.1)	98.3	93.6	71.7	
Group 4 (n=16)	9 (56.3)	46.9	37.5	25	
CHP					
Total (N=299)	25 (8.4)	94.2	80.3	74.7	
Group 1 (n=194)	11 (5.7)	98.2	85.8	82.1	<0.001
Group 2 (n=6)	3 (50)	66.7	0		
Group 3 (n=94)	7 (7.4)	90.9	75	62.5	
Group 4 (n=5)	0 (0)	0			
NS					
Total (N=89)	6 (6.7)	95.2	93	85.3	
Group 1 (n=61)	0 (0)				<0.001
Group 2 (n=6)	0 (0)				
Group 3 (n=15)	2 (13.3)	77.8			
Group 4 (n=7)	4 (57.1)	71.4	47.6	0	

Group 1: Remission and eGFR at renal biopsy ≥60
Group 2: Remission and eGFR at renal biopsy <60
Group 3: No remission and eGFR at renal biopsy ≥60
Group 4: No remission and eGFR at renal biopsy <60

CKD; chronic kidney disease stage 3–5, GHU; gross hematuria, CHP; combined hematuria with proteinuria, NS; nephrotic syndrome
